# Microstates as Disease and Progression Markers in Patients With Mild Cognitive Impairment

**DOI:** 10.3389/fnins.2019.00563

**Published:** 2019-06-11

**Authors:** Christian Sandøe Musaeus, Malene Schjønning Nielsen, Peter Høgh

**Affiliations:** ^1^Department of Neurology, Danish Dementia Research Centre, Rigshospitalet, University of Copenhagen, Copenhagen, Denmark; ^2^Regional Dementia Research Centre, Department of Neurology, Zealand University Hospital, Roskilde, Denmark; ^3^Department of Clinical Medicine, University of Copenhagen, Copenhagen, Denmark

**Keywords:** EEG, mild cognitive impairment, Alzheimer, Alzheimer’s disease, progression, stable, MCI, microstate

## Abstract

Network dysfunction is well established in patients with Alzheimer’s disease (AD) and has been shown to be present early in the disease. This is especially interesting in patients with mild cognitive impairment (MCI) since they are more likely to develop AD. In EEG, one type of network analysis is microstates where the EEG is divided into quasi-stable states and these microstates have been linked to networks found with resting state functional MRI. In the current exploratory study, we therefore wanted to explore the changes in microstates in MCI, and AD compared to healthy controls (HC) and whether microstates were able to separate patients with MCI who progressed (pMCI) and those who remained stable (sMCI). EEGs were recorded at baseline for 17 patients with AD, 27 patients with MCI, and 38 older HC and the patients were followed for 3 years. To investigate whole-brain dynamics we extracted different microstate parameters. We found that patients with MCI, and AD had significantly higher occurrence (*p*-value = 0.028), and coverage (*p*-value = 0.010) for microstate A compared to HC. However, we did not find any significant systematic deviation of the transition probabilities from randomness for any of the groups. No significant differences were found between pMCI and sMCI but the largest difference in duration was found for microstate D. Microstate A has been linked to the temporal lobes in studies combining EEG and fMRI and the temporal lobes are the most affected by AD pathology in the early stages of the disease. This supports our idea that microstate A may be the first affected microstate in early AD. Even though not significant between pMCI and sMCI, Microstate D has previously been shown to be associated with both frontal and parietal areas as measured with fMRI and may correspond to underlying pathological changes in the progression of MCI to AD. However, larger studies are needed to confirm these findings.

## Introduction

Alzheimer’s disease (AD) is a progressive neurodegenerative disease and patients with AD have shown changes in functional brain networks (Dickerson and Sperling, 2009). Studies have even suggested that alterations in networks are present very early in the disease process (Selkoe, 2002; Cummings, 2004). Patients mild cognitive impairment (MCI), which is thought of as mild objective cognitive deficits, are associated with later development of AD (Petersen et al., 1999; Petersen, 2004). While some patients with MCI progress (pMCI) others remain stable (sMCI) in their disease, which is in large due to different etiological causes as for example depression or vascular changes. However, for patients with MCI due to AD, there is also evidence of fast and slow progression (Chui, 1987), which may be due to affection of different brain networks.

Multiple methods to investigate brain networks have been proposed with the most common being fMRI. But since network function are thought of as fast processes that changes over time, fMRI may not be able to capture these. Electroencephalography (EEG) has a high temporal resolution and methods like microstate analysis has been able to show topographical maps that have been associated with resting state networks (Van de Ville et al., 2010; Yuan et al., 2012). Microstates is a technique where the multichannel resting-state EEG signal can be divided into a number of distinct states (Lehmann et al., 1987). Although these states occur in a time range of milliseconds (ms), it has been shown that momentary stable spatial patterns that last approximately 80–120 ms before rapidly transitioning to a different microstate (Khanna et al., 2015). The majority of the studies have clustered the resting EEG into four microstate classes, which has been found to be the optimal number according to cross-validation criterion (Pascual-Marqui et al., 1995; Koenig et al., 2002) and a study found a high test-retest reliability (Khanna et al., 2014).

Only few studies have investigated alterations in microstates in patients with AD (Ihl et al., 1993; Dierks et al., 1997; Strik et al., 1997; Stevens and Kircher, 1998; Nishida et al., 2013). Most studies found a shorter duration of the microstates compared to healthy elderly controls with one study finding a longer duration of microstates. However, a more recent study has found no significant changes in either duration or occurrence in patients with AD compared to healthy controls (Nishida et al., 2013). Moreover, none of the studies have investigated the early changes in microstates by looking at patients with MCI or whether microstates are able to differentiate between pMCI and sMCI.

In the current exploratory study, we wanted to investigate the changes in microstates in patients with MCI compared to both AD, and healthy controls (HC). Furthermore, we wanted to investigate whether microstates can be used to separate pMCI from sMCI. Lastly, we wanted to investigate whether any microstates parameters correlated with either cognitive scores or AD biomarkers.

## Materials and Methods

### Recruitment, Inclusion Criteria, and Subjects

The whole dataset or parts of the dataset have also been used for other studies (Engedal et al., 2015; Musaeus et al., 2018a, 2019; Nielsen et al., 2018) including separate presentation of results from spectral power analysis (Musaeus et al., 2018b) and for coherence and imaginary part of coherency analysis (Musaeus et al., 2019).

This prospective cohort study was conducted at two Danish memory clinics at Zealand University Hospital and Rigshospitalet, respectively. Patients consecutively referred for cognitive evaluation and diagnosed with either MCI or mild AD and at least a baseline Mini-Mental State Examination (MMSE) score of ≥22 were eligible for inclusion. The patient selection was defined using preexisting exclusion criteria: (1) no close relatives who wished to participate, (2) if they were participating in other intervention studies or (3) if they were suffering from other neurological, psychiatric, or other severe disease, (4) if they received sedative medication due to a potential sedative effect, and (5) if they had any past or current addictions to alcohol or medications.

The HC were all volunteers recruited trough public advertisements at the memory clinics, at local associations for elders and through an online recruitment site for trial subjects. Inclusion criteria were: (1) age between 50 and 90 years, (2) MMSE score ≥26, (3) ACE ≥85, (4) normal neurological and clinical examination, (5) normal or age-related brain atrophy measured on a computed tomography (CT) scan, (6) normal routine blood tests. Exclusion criteria were: (1) an inability to participate (including impaired vision or hearing), (2) presence of cognitive symptoms including memory complaints, (3) signs of major neurological, psychiatric or other severe disease, which potentially could elicit cognitive impairments including signs of major depression or a geriatric depression scale score >7, (4) be pregnant, (5) have undergone general anesthesia, (6) received electroconvulsive therapy in the past 3 months, (7) receive sedatives, or (8) have any past or current addictions to alcohol or medications.

In total, we included 17 patients with AD, 27 patients with MCI, and 38 HC. The study was reported to and approved by the Danish Data Protection Agency and by the Regional Ethical Committee according to Danish legislation.

### Diagnostic Assessment

The patients underwent a standardized diagnostic assessment including a full physical and neurological examination, routine blood analysis, brain CT or MRI scan as well as cognitive screening, i.e., MMSE, Addenbrooke’s Cognitive Examination (ACE), Digit Symbol Substitution Test (DSST), and Clinical Dementia Rating (CDR). Furthermore, as part of the diagnostic assessment patients and relatives underwent NeuroPsychiatric Inventory (NPI), Major Depression Inventory (MDI), Activities of Daily Living Inventory (ADCS-ADL). The CT and MRI scans were examined by a neuro-radiologist. The majority also had a lumbar puncture (except two patients with MCI and six HC) performed to measure AD biomarkers (Amyloid-β_42_, total tau, and phosphorylated tau), and for routine parameter analysis. If diagnostically relevant, the patients also had a neuropsychological evaluation undertaken by a clinical neuropsychologist, but these were individualized for each patient with varying overlap and therefore not included in the current study. Diagnoses were settled by consensus of a multidisciplinary team based on all examination results. The included MCI patients fulfilled the Winblad consensus criteria (Winblad et al., 2004) and AD patients fulfilled the NIA-AA criteria (McKhann et al., 2011).

At inclusion, all HC underwent the standardized diagnostic assessment, which included cognitive tests (ACE, MMSE, DSST), MDI and analysis of CSF was performed on almost all HC. At the baseline visit all HC were referred for a standardized EEG. The EEG recordings were not used in the diagnostic assessment.

### Study Design

The patients were recruited within 6 months after the diagnosis and all tests were repeated at inclusion. Follow-up visits were carried out on a yearly basis, with serial cognitive tests, i.e., MMSE and ACE and the NPI, MDI, ADCS-ADL, and CDR scales. Clinical progression of MCI to AD was determined based on whether the patient clinically fulfilled the NIA-AA criteria (McKhann et al., 2011). If the patient progressed to another diagnosis, they were excluded from the comparison between pMCI and sMCI.

The primary investigator performing the tests was blinded for the results of the EEG, imaging and CSF analysis during the study period. This was done for the investigator to be blinded for the potential presence of underlying AD pathology.

### Electroencephalography Recording

The EEG recordings were performed at the two participating centers and the EEG recordings were performed using NicoletOne EEG Systems (Natus^®^) with a sampling rate of either 500 or 1000 Hz. Nineteen electrodes were positioned according to the International 10–20 system. Most EEGs were recorded with alternating eyes closed (EC) and eyes open periods for 3 min each but some of the recording only had EC segments. The participants were alerted if they became visibly drowsy, since drowsiness influences recording. The neurophysiology assistant recording the EEG made marks in the EEG when the participant closed and open their eyes. After the recording, the files were exported as raw EEGs without any filtering.

### Collection and Analysis of Cerebrospinal Fluid

The lumbar puncture was performed between the L3/L4 or L4/L5 intervertebral space and the CSF was collected in polypropylene tubes. Analysis of the CSF included routine parameters and the core AD biomarkers, i.e., Aβ_42_, T-tau, and P-tau. The AD biomarkers were quantified with sandwich ELISAs [INNOTEST amyloid-β_42_, hTau, and Phospho-Tau (181P), respectively; Fujirebio Europe, Ghent, Belgium]. AD biomarkers analyses from both clinics were all carried out at one central laboratory.

### Preprocessing of EEG

The EEG data were imported to MATLAB (Mathworks, v2016a) using the EEGLAB toolbox (Delorme and Makeig, 2004). Only segments with EC were selected either using markers placed doing recording or from the first 10 min of recording if markers were not present. The electrodes were computationally located on the scalp using the dipfit toolbox (Oostenveld et al., 2011) with the standard 10–20 electrode model. The excessive channels were removed, and the data were bandpass filtered from 1 to 70 Hz using the *pop_firws* function in MATLAB with a filter order of 2 and the Kaiser window parameter beta was estimated using a maximum passband ripple of 0.001. Furthermore, the data were bandstop filtered from 45 to 55 Hz using the same settings as described previously. Afterward, the data were down sampled to 200 Hz. Then, the data were divided into 1 s epochs and the EEGs were visually inspected and epochs with excessive noise or artifacts were removed. Channels with excessive noise, drift, or bad connection were interpolated using spherical interpolation. The EEG had to have ≤ three electrodes with excessive artifact, otherwise the EEG was excluded from the analysis. Afterward, the EEGs were re-referenced to average and independent component analysis (ICA) was performed using the extended infomax algorithm (Lee et al., 1999) for each file and components that contained eye blinks, eye movement, or specific line noise artifacts were removed manually. Lastly, the EEGs were inspected visually again and epoch with excessive noise or artifacts were removed. The investigator who performed the preprocessing was blinded to the diagnosis. Due to excessive artifacts, we excluded the following number of EEGs: two from patients with AD, two from patients with MCI, and one from HC. When comparing pMCI, and sMCI, one EEG from MCI was excluded due to clinical progression to vascular dementia.

### Microstate Analysis

**FIGURE 1:**
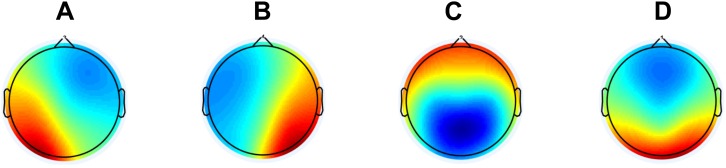
The global maps that were calculated based the aggregated dataset from all participants and were back-fitted to each of the EEG recordings. The labels **(A–D)** are according to the previous literature in the field.

Before performing the microstate analysis, we first lowpass filtered the data at 20 Hz with the same settings as mentioned above. Afterward, we concatenated the epochs for each subject, i.e., ending up having one continuous EEG file. We performed the microstates analysis using the *Microstate EEGlab Toolbox* (Poulsen et al., 2018). Here, we first extracted the global field power (GFP) peaks and the settings were a minimum peak distance of 10 ms, the number of GFP peaks per subject that enter the segmentation was set at 1000, and GFP peaks that exceeded 2 times the standard deviation of the GFPs of all maps were excluded. All the GFP peaks from all subjects were aggregated into one file before segmentation with the goal to maximizing the similarity between the microstates they would be assigned to. For segmentation, we used the modified K-means algorithm since it ignores the polarity of the EEG topography (Lehmann, 1971; Wackermann et al., 1993; Pascual-Marqui et al., 1995). Here, we predefined the number of microstates as four, which previously has been reported as the most common (Khanna et al., 2014) and reproduceable (Khanna et al., 2015). The number of repetitions were set at 50 and maximum number of iterations were set at 1000. The global maps (see **Figure 1**) were then back-fitted to each of the EEG files by labeling each of EEG segments with the class of microstates it is most familiar. Since resting state EEG is noisy, it happens that consecutive time frames are labeled different by change. To avoid this, we rejected microstate segments shorter than 30 ms. The labels of time frames in small segments were changed to the next most likely microstate class, as measured by global map dissimilarity (Poulsen et al., 2018). After back-fitting the global maps, we calculated global explained variance (GEV), duration, occurrence, coverage, and the syntax for EEG files.

As a *post hoc* examination of the transition probabilities, we performed the same analysis as previously described in detail (Lehmann et al., 2005; Nishida et al., 2013). In short, we calculated the observed transitions based on all transitions and then the expected transitions based on the occurrence of the microstates for each subject separately. Afterward, these values (4 × 4 - 4 = 12) were averaged across subjects for each group, and the difference was assessed using the chi-square distance. To statistically test the difference, we performed a permutation test with 5000 repetitions where the labels “expected” and “observed” were randomly assigned to the subjects’ sets of the 12 transition probabilities, and the chi-square distance was computed. The underlying hypothesis of this test was that if transitions from one state into the next occurred randomly, observed transition values would be proportional to the relative occurrence of the microstate classes.

### Statistics

MATLAB (vR2016a) was used for all statistical analyses. When comparing demographics, number of epochs, average GEV and cognitive scores for AD, MCI, and HC, we performed one-way ANOVAs. Independent *t*-tests were used to compare baseline cognitive scores between pMCI and sMCI. Since the microstate results (duration, occurrence, coverage, and syntax) were non-normally distributed, we log-transformed the data. Afterward, we performed an ANCOVA with age, gender, education, and current medication (see **Table 1**) as covariates. If we found a significant difference (*p*-value < 0.05), we performed independent *t*-tests (without covariates) between AD vs. HC, MCI vs. HC, and AD vs. MCI. For the microstate results from baseline EEG between pMCI vs. sMCI we used an ANCOVA with the same covariate as mentioned above. The division of the baseline EEGs into either pMCI and sMCI was determined on progression after 2nd year follow-up. We used the data before long-transformation for display in the tables. For the *post hoc* analyses between microstate features, we also calculated effect size measured with Cohen’s d, which is defined as the difference between two means divided by the standard deviation of the data.

**Table 1:** Table showing the characteristics of the participants included in the analysis.

	HC (*n* = 37)	AD (*n* = 15)	MCI (*n* = 25)	*p*-value
Mean age (SD), years	65.7 (6.9)	70.1 (7.8)	71.4 (6.0)	0.006
Female gender, n	17	8	6	0.119
Education, years (SD)	12.7 (3.6)	12.1 (4.0)	10.6 (3.4)	0.105
MMSE, mean (SD)	29.1 (1.0)	26.3 (3.2)	27.6 (1.5)	0.001*
Antidepressants	1	1	4	0.161
Cholinesterase Inhibitors	0	8	1	0.001*
Pain killers	2	0	2	0.553
CSF amyloid, mean (SD)	997.5 (320.2)	550.7 (141.2)	782.3 (319.8)	0.001*
CSF total tau, mean (SD)	303.3 (144.7)	618.4 (186.0)	419.6 (173.9)	0.001*
CSF phosphorylated tau, mean (SD)	68.5 (103.4)	93.0 (33.3)	59.4 (21.5)	0.384
EEG length, mean seconds (SD)	177.5 (62.1)	147.1 (19.6)	153.6 (44.6)	0.078

HC, healthy controls; AD, Alzheimer’s disease; MCI, mild cognitive impairment; SD, standard deviation; MMSE, Mini-Mental State Examination; CSF, cerebrospinal fluid. *Indicates significant p-value (< 0.05).

Furthermore, we performed Spearman’s correlation using the values from significant differences between AD, MCI, and HC (coverage, and occurrence for microstate A). We chose to correlate these values with amyloid, total tau, phosphorylated tau, MMSE, and ACE.

## Results

### Demographics, Cognitive Tests, and EEG Length

**FIGURE 2:**
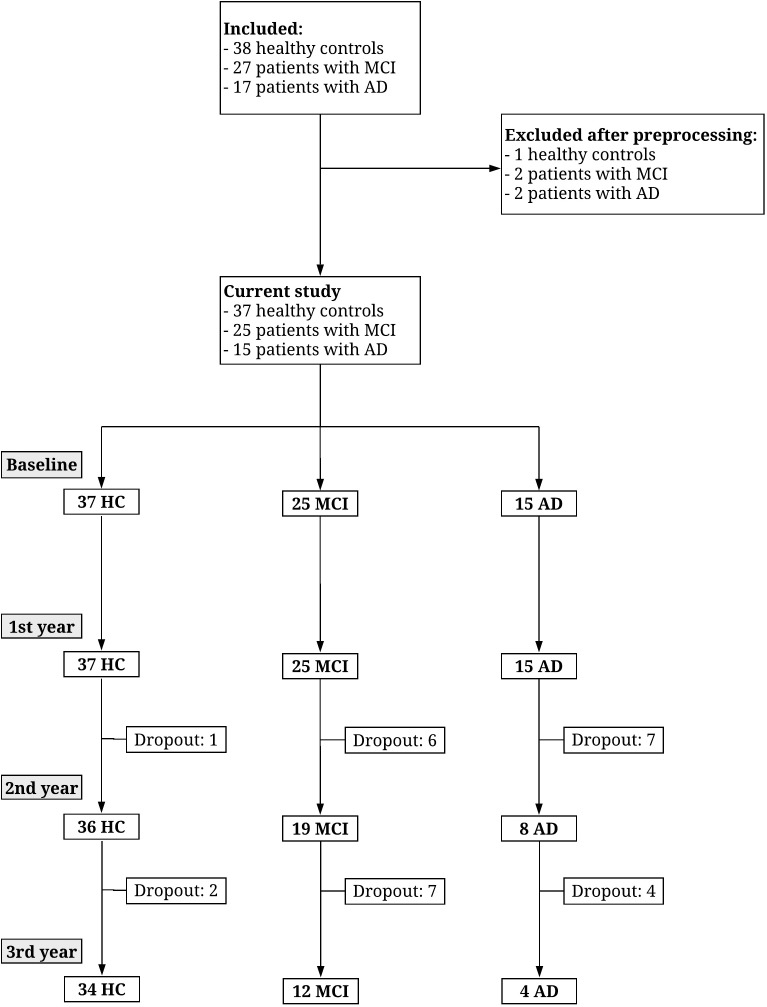
Flow diagram showing the number of participants recruited and drop-out over 3 years. Figure reproduced from Musaeus et al. (2018b).

Characterization of the patients including cognitive test scores and EEG length is shown in **Table 1**. For the performance on cognitive tests for each visit see **Table 2**. For the comparison between demographics, baseline cognitive scores, and CSF biomarkers for pMCI and sMCI see **Table 3**. Flow diagram of the included patients is illustrated in **Figure 2**.

**Table 2:** The cognitive scores, number of participants that dropped out, and number of patients with MCI that progressed doing follow-up for year 2.

		Baseline	2nd year follow-up	*t*-value	*p*-value
HC	Dropout/total (n)	0	1/37		
	Progression/no-progression	NR	NR		
	MMSE, mean (SD)	29.08 (0.98)	29.36 (0.83)	-1.312	0.194
	ACE, mean (SD)	94.70 (3.28)	95.58 (3.32)	-1.139	0.259
	MDI, mean (SD)	3.62 (2.87)	4.06 (3.30)	-0.600	0.551
MCI	Dropout/total (n)	0	6/25		
	Progression/no-progression	NR	12/13		
	MMSE, mean (SD)	27.60 (1.50)	26.00 (3.33)	2.138	0.038*
	ACE, mean (SD)	84.13 (8.17)	79.67 (11.59)	1.464	0.151
	MDI, mean (SD)	7.13 (5.91)	10.22 (7.75)	-1.450	0.155
	NPI, mean (SD)	3.38 (3.49)	5.24 (2.49)	-1.844	0.073
	ADL, mean (SD)	70.71 (4.84)	66.59 (9.87)	1.544	0.133
AD	Dropout/total (n)	0	7/15		
	Progression/no-progression	NR	NR		
	MMSE, mean (SD)	26.27 (3.17)	23.50 (5.53)	1.537	0.139
	ACE, mean (SD)	77.60 (12.87)	67.14 (18.85)	1.532	0.141
	MDI, mean (SD)	5.67 (4.70)	4.17 (4.62)	0.664	0.515
	NPI, mean (SD)	1.5 (1.24)	5.00 (2.45)	-4.235	< 0.000*
	ADL, mean (SD)	70.86 (8.16)	67.38 (8.67)	0.942	0.358
	Missing values (%)	6.78	26.21		

In addition, the percentage of missing values for the cognitive scores can be seen. All cognitive scores have been compared over time using a paired t-test. *Indicates significant p-value ( < 0.05).

**Table 3:** Demographics, baseline cognitive scores, and CSF results for stable mild cognitive impairment (sMCI) and progressed mild cognitive impairment (pMCI).

	Baseline – sMCI (*n* = 13)	Baseline – pMCI (*n* = 11)	*p*-value
Mean age (SD), years	72.38 (6.06)	70.27 (6.63)	0.424
Female gender, n	4	2	0.500
Education, years (SD)	10.69 (3.84)	10.55 (3.36)	0.922
CSF amyloid, mean (SD)	820.08 (348.64)	695.75 (309.90)	0.419
CSF total tau, mean (SD)	398.25 (162.10)	461.56 (206.29)	0.440
CSF phosphorylated tau, mean (SD)	60.54 (24.54)	59.89 (19.28)	0.948
MMSE, mean (SD)	27.92 (1.38)	27.09 (1.58)	0.182
ACE, mean (SD)	87.54 (6.08)	79.00 (8.36)	0.010*
MDI, mean (SD)	8.67 (6.89)	6.00 (4.14)	0.297
NPI, mean (SD)	3.09 (3.96)	3.00 (2.24)	0.952
CDR, mean (SD)	0.50 (0)	0.56 (0.17)	0.281
ADL, mean (SD)	70.60 (6.06)	70.86 (2.73)	0.918

T-tests were performed to compare the two groups for each score separately. *Indicates significant (p-value < 0.05) difference. One patient with MCI showed up during follow-up to fulfill the criteria for vascular dementia and was not included in the comparison between pMCI and sMCI.

### Microstates Results Between HC, MCI, and AD

The average GEV was not significantly different between HC (mean = 0.54, *SD* = 0.08), MCI (mean = 0.53, *SD* = 0.10), and AD (mean = 0.56, *SD* = 0.03), (*p*-value = 0.3624, *F*-value = 1.0290).

See **Figure 1** for global maps of the microstates that were used for back-fitting. Between AD, MCI, and HC, we found significantly different occurrence (*p*-value = 0.0277, *F*-value = 3.7807, degrees of freedom = 68) and coverage (*p*-value = 0.0101, *F*-value = 4.9237, degrees of freedom = 68) for microstate A, see **Table 4**. For the *post hoc t*-test for occurrence we found a significant difference between AD, and HC (*p*-value = 0.0395, *t*-value = 2.1142, Cohen’s *d* = 0.6471) and between MCI, and HC (*p*-value = 0.0411, *t*-value = 2.0874, Cohen’s *d* = 0.5404). For coverage, we found a significant difference between AD, and HC (*p*-value = 0.0066, *t*-value = 2.8359, Cohen’s *d* = 0.8681) and between MCI, and HC (*p*-value = 0.0077, *t*-value = 2.7575, Cohen’s *d* = 0.7139).

**Table 4:** Table showing the mean, standard deviation (SD), and *p*-value for comparisons between the three groups (HC, MCI, and AD) for microstates A–D for duration, occurrence, and coverage.

	Duration				Occurrence				Coverage			
	HC	MCI	AD	*p*-value	HC	MCI	AD	*p*-value	HC	MCI	AD	*p*-value
Microstate A, (*SD*)	75.88 (10.05)	82.23 (11.05)	83.19 (11.05)	0.059	2.45 (0.65)	2.78 (0.55)	2.83 (0.45)	0.028	0.19 (0.06)	0.23 (0.06)	0.24 (0.04)	0.01
Microstate B, (*SD*)	78.10 (12.42)	85.45 (11.48)	78.62 (9.15)	0.082	2.56 (0.87)	2.81 (0.67)	2.62 (0.68)	0.422	0.21 (0.09)	0.24 (0.08)	0.21 (0.07)	0.26
Microstate C, (*SD*)	89.08 (23.19)	90.76 (42.38)	87.12 (16.95)	0.725	2.98 (0.76)	2.61 (0.59)	3.01 (0.56)	0.256	0.27 (0.11)	0.25 (0.15)	0.26 (0.8)	0.578
Microstate D, (*SD*)	106.02 (41.73)	92.26 (16.32)	94.32 (22.69)	0.55	3.16 (0.63)	2.93 (0.66)	3.09 (0.41)	0.304	0.34 (0.13)	0.28 (0.09)	0.29 (0.08)	0.307

**FIGURE 3:**
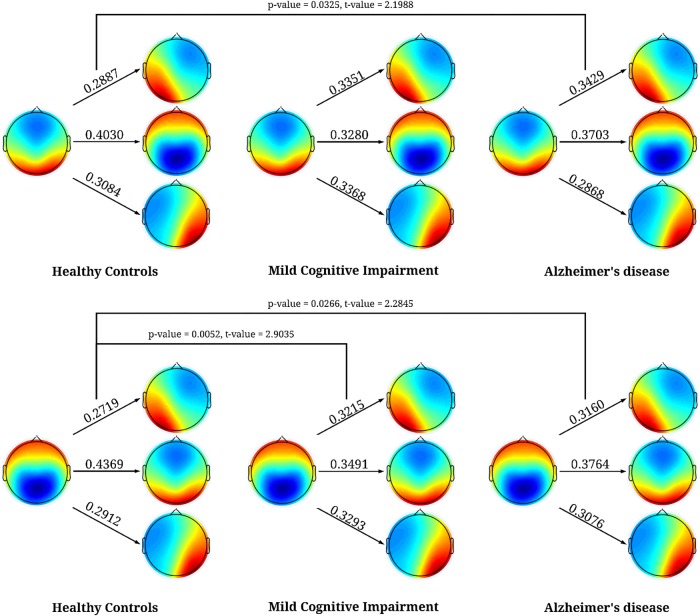
Significant results for the syntax analysis between HC, MCI, and AD. The first column is for HC, second for MCI, and third for AD. The values represent the percentage of times when microstates C, and D transitioned to the other microstates. The figure shows that both microstates C, and D were more likely to transition to microstate A in patients with AD and in patients with MCI microstate C transitioned significantly more to microstates A.

For the syntax analysis, we found patients with MCI and AD were significantly more likely to transition from microstates C to A, and for AD from and D to A compared to HC when only looking at the observed transition percentages for each microstate separately, see **Figure 3**. However, when we performed the permutation test between observed and expected percentage of transitions, we did not find any systematic deviation of transition from randomness (*p*-value > 0.05). See **Table 6** for observed and expected percentage of transitions.

### Microstates Between pMCI and sMCI

No significant differences (*p*-value < 0.05) were found between pMCI and sMCI for duration, occurrence, or coverage. The largest difference in duration was found for microstate D between pMCI and sMCI (see **Table 5**).

**Table 5:** Table showing the mean, standard deviation (SD), and *p*-value for comparisons between pMCI, and sMCI for microstates A-D for duration, occurrence, and coverage.

	Duration			Occurrence			Coverage		
	pMCI	sMCI	*p*-value	pMCI	sMCI	*p*-value	pMCI	sMCI	*p*-value
Microstate A, (SD)	75.32 (8.98)	76.22 (10.48)	0.594	2.41 (0.67)	2.47 (0.53)	0.587	0.18 (0.06)	0.19 (0.05)	0.547
Microstate B, (SD)	79.32 (16.66)	76.29 (8.49)	0.296	2.41 (0.96)	2.62 (0.84)	0.654	0.20 (0.12)	0.20 (0.08)	0.463
Microstate C, (SD)	81.59 (18.26)	95.06 (22.59)	0.673	2.61 (0.74)	3.31 (0.48)	0.708	0.22 (0.11)	0.31 (0.07)	0.655
Microstate D, (SD)	116.90 (33.91)	93.08 (26.15)	0.235	3.30 (0.58)	3.15 (0.75)	0.292	0.39 (0.14)	0.30 (0.10)	0.238

**Table 6:** Observed and expected percentage of transitions.

	HC		MCI		AD	
	Observed	Expected	Observed	Expected	Observed	Expected
D to C	0.118	0.111	0.084	0.084	0.1	0.096
D to A	0.083	0.088	0.089	0.089	0.094	0.092
D to B	0.087	0.089	0.088	0.089	0.076	0.081
C to D	0.119	0.108	0.085	0.083	0.099	0.095
C to A	0.072	0.079	0.078	0.079	0.082	0.086
C to B	0.076	0.081	0.077	0.078	0.08	0.079
A to D	0.084	0.08	0.088	0.087	0.092	0.088
A to C	0.074	0.074	0.077	0.077	0.084	0.084
A to B	0.062	0.066	0.085	0.085	0.069	0.073
B to D	0.086	0.082	0.089	0.087	0.079	0.077
B to C	0.075	0.076	0.079	0.078	0.076	0.076
B to A	0.065	0.067	0.082	0.085	0.07	0.072

We also performed syntax analysis, but no significant differences were found between pMCI and sMCI.

### Correlation

No significant correlations were found between coverage, and occurrence for microstates A and amyloid, total tau, phosphorylated tau, MMSE, or ACE.

## Discussion

In the current exploratory study, we found that patients with MCI, and AD compared to HC had significantly higher occurrence and coverage of microstate A. In addition, both microstates C and D transitioned significantly more to microstate A in patients with AD compared to HC, and microstate C transitioned more to microstate A in MCI compared to HC. However, we did not find evidence that there was any systematic deviation of transition probabilities from randomness for any of the groups. Between pMCI, and sMCI, we did not find any significant differences but the largest difference in duration was found for microstate D. Lastly, no correlations were found between microstate A and either biomarkers or neuropsychological tests.

Previous studies have investigated patients with AD (Ihl et al., 1993; Dierks et al., 1997; Strik et al., 1997; Stevens and Kircher, 1998; Nishida et al., 2013) but the majority found a shorter duration of the microstates in patients suffering from AD (Dierks et al., 1997; Strik et al., 1997; Stevens and Kircher, 1998) compared to healthy older controls. In the early studies (Ihl et al., 1993; Dierks et al., 1997; Strik et al., 1997; Stevens and Kircher, 1998) adaptive segmentation was used, which may have given rise to different results. However, a more recent study using clustering analysis (Nishida et al., 2013) did not find any significant differences between patients with AD and HC, which could be due to low sample size or as previously suggested temporal disorganization in patients with AD (Koenig et al., 2005; Nishida et al., 2013). However, in the current study we found longer duration in both patients with MCI, and AD with significant increased occurrence and coverage in microstate A compared to HC, see **Table 4**. The main reason for our finding compared to the recent study not finding any significant results between AD, and HC (Nishida et al., 2013) may be differences in methods. Here, we compiled GFP peaks for all participants before segmentation or it could be due to differences in the recruitment. However, significantly increased occurrence and coverage for microstate A has not previously been reported and the underlying reason may be underlying AD pathology in the temporal lobes, which has been shown in pathological studies using Braak staging (Braak and Braak, 1991; Thal et al., 2002) and studies using follow-up data on the deposition of amyloid with PiB-PET (Okello et al., 2009; Villemagne et al., 2011). The increased coverage and occurrence of microstate A may therefore be due to underlying pathological changes in the temporal lobes and thereby disruption of the underlying neuronal networks. Interestingly, no statistically significant differences were found between MCI, and AD, which may indicate that the majority of the included patients with MCI had an underlying AD pathology. Supportive of this assumption, is the observation that more than half of the MCI cohort progressed significantly clinically over 2 years follow-up. However, we did not find any significant changes in the microstate B between AD, MCI, and HC, which may be due the topographical map did not involve as large a region of the temporal region as microstate A. However, in a previous paper using the same data for spectral power analysis, we found that the changes are more pronounced on the left side (Musaeus et al., 2018b). This effect may correspond to previous MR studies showing atrophy being more pronounced on the left side (Killiany et al., 2000; Baron et al., 2001) or a previous study showing more pronounced hypometabolism in the left temporal region using SPECT (Hogh et al., 2004). On the other hand, this may also simply be due to low sample size and thereby individual differences affecting the results. For the syntax analysis, we found that patients with both AD, and MCI were more likely to transition from microstate C to A, and AD from D to A, see **Figure 3**. However, we did not find any systematic deviation of transition probabilities from randomness, which strongly indicates that the transitions were in large part due to the increased occurrence of microstate A.

The microstate classes have also been associated with BOLD signal and resting state networks obtained with fMRI in multiple studies (Britz et al., 2010; Van de Ville et al., 2010; Yuan et al., 2012). One study has associated microstate A with BOLD activations in the superior and middle temporal gyri as well as the left middle frontal gyrus (Britz et al., 2010). Other studies extracted 13 (Yuan et al., 2012) and 10 (Musso et al., 2010) microstates, respectively, and any direct comparisons were therefore very difficult. By visual inspection, it is possible that microstate A may correspond to microstate 5 and 13 in a previous publication (Yuan et al., 2012) and thereby be associated with the default mode network. These findings suggest that microstate A is associated with temporal connectivity and may even be related to the default mode network.

In patients with pMCI and sMCI, we did not find any significant changes but the largest difference in duration was found for microstate D, see **Table 5**. Microstate D has previously been associated with BOLD changes in the frontal and parietal areas measured with fMRI (Britz et al., 2010) and may reflect underlying pathological changes in patients with MCI who progress to AD. However, larger studies are needed to test whether microstate D is in fact different between pMCI and sMCI.

Previous studies have found an inverse correlation between microstate lifespan and degree of cognitive impairment (Dierks et al., 1997; Strik et al., 1997). In the current study, we did not find any correlation between occurrence or coverage and either biomarkers or neuropsychological tests. This may be due to the low sample size or the values extracted based on the global maps. Larger studies are needed to investigate whether microstate changes are associated with neuropsychological findings.

In the current study, we choose to extract four microstates since this is the most commonly reported and these have been shown to be reliable (Khanna et al., 2014). However, the GEV was not significantly different between the three groups but was low (average GEV = 54%) compared to other studies with most commonly reporting a GEV >70% (Michel and Koenig, 2018). The low GEV may be due to either broad filter settings (2–20 Hz) or simply due to patient data being noisier. In the current analysis, we included only the first 1000 GFP peaks to the segmentation and thereby avoided problems in terms of more contributions from larger EEG files.

The study indicates that microstate A could be an early disease marker in patients with MCI, but it has some limitations. Firstly, we acknowledge the relatively small sample size and we did not correct for multiple comparisons due to the exploratory nature of the study. However, these changes suggest that larger studies will be able to use microstates as a classifier of disease even at an early stage. In addition, the follow-up time was short and according to previous studies, annual clinical progression rate is 15% (Petersen et al., 1999; Saxton et al., 2009), which means that only 30% of the patients with MCI should have progressed to AD. However, we found that 48% progressed, which may in part be due to the patients with MCI being at a more advanced stage of the disease at inclusion. Furthermore, we included patients receiving medication in the analysis, which may have affected the EEG. Nevertheless, our findings in this small pilot study with affected microstate A in patients with MCI and possible affection of microstate D in the transition from MCI to AD may be able to guide larger studies.

## Conclusion

In the current exploratory study, we found that patients with MCI, and AD compared to HC had significantly higher occurrence and coverage of microstate A. The changes may correspond to the previous literature of pathological changes in the temporal regions in patients with AD and microstate A may correspond to temporal regions measured with BOLD fMRI. Furthermore, between pMCI, and sMCI, no significant differences were found but a tendency of a prolonged duration of microstate D in patients with pMCI was seen. Larger studies are needed to confirm these findings.

## Data Availability

The datasets supporting the conclusions of this manuscript will be made available by the authors to any qualified researcher. However, due to regulations, we are not able to share the EEG files.

## Ethics Statement

This study was carried out in accordance with the recommendations of the Regional Committee on Health Research Ethics with written informed consent from all subjects. All subjects gave written informed consent in accordance with the Declaration of Helsinki. The protocol was approved by the Regional Committee on Health Research Ethics.

## Author Contributions

PH, MN, and CM conceived the project idea of using quantitative EEG. PH and MN conducted the experiments. CM conducted the data analyses and drafted the manuscript. PH, MN, and CM contributed to revising the manuscript.

## Conflict of Interest Statement

The authors declare that the research was conducted in the absence of any commercial or financial relationships that could be construed as a potential conflict of interest.
